# Changes in Growth and Heavy Metal and Phenolic Compound Accumulation in *Buddleja cordata* Cell Suspension Culture under Cu, Fe, Mn, and Zn Enrichment

**DOI:** 10.3390/plants13081147

**Published:** 2024-04-19

**Authors:** Alicia Monserrat Vazquez-Marquez, Antonio Bernabé-Antonio, José Correa-Basurto, Cristina Burrola-Aguilar, Carmen Zepeda-Gómez, Francisco Cruz-Sosa, Aurelio Nieto-Trujillo, María Elena Estrada-Zúñiga

**Affiliations:** 1 Facultad de Ciencias, Universidad Autónoma del Estado de México, Campus El Cerrillo, Piedras Blancas, Carretera Toluca-Ixtlahuaca Km. 15.5, Toluca CP 50200, México; 2 Departamento de Madera, Celulosa y Papel, Centro Universitario de Ciencias Exactas e Ingenierías, Universidad de Guadalajara, Km. 15.5, Carretera Guadalajara-Nogales, Col. Las Agujas, Zapopan CP 45200, México; 3 Escuela Superior de Medicina, Instituto Politécnico Nacional, Plan de San Luis y Díaz Mirón S/N, Casco de Santo Tomas, Miguel Hidalgo, Ciudad de México CP 11340, México; 4 Centro de Investigación en Recursos Bióticos, Facultad de Ciencias, Universidad Autónoma del Estado de México, Carretera Toluca-Ixtlahuaca Km 14.5, San Cayetano, Toluca CP 50295, México; 5 Departamento de Biotecnología, Universidad Autónoma Metropolitana-Iztapalapa, Av. Ferrocarril San Rafael Atlixco No. 186, Col. Leyes de Reforma 1a. Sección, Alcaldía Iztapalapa, Ciudad de México CP 09310, México

**Keywords:** heavy metal tolerance, elicitation, secondary metabolite, verbascoside

## Abstract

*Buddleja cordata* cell suspension cultures could be used as a tool for investigating the capabilities of this species to tolerate heavy metals (HMs) and for assessing the effects of HMs on the accumulation of phenolic compounds in this species. It grows in a wide range of habitats in Mexico, including ultramafic soils, and mobilizes some HMs in the soil. The mobilization of these HMs has been associated with phenolic substances. In addition, this species is used in Mexican traditional medicine. In the present study, a *B. cordata* cell suspension culture was grown for 18 days in a culture medium enriched with Cu (0.03–0.25 mM), Fe (0.25–1.5 mM), Mn (0.5–3.0 mM), or Zn (0.5–2.0 mM) to determine the effects of these HMs on growth and HM accumulation. We also assessed the effects of the HMs on phenolic compound accumulation after 1 and 18 days of HM exposure. Cells were able to grow at almost all tested HM concentrations and accumulated significant amounts of each HM. The highest accumulation levels were as follows: 1160 mg Cu kg^−1^, 6845 mg Fe kg^−1^, 3770 mg Mn kg^−1^, and 6581 mg Zn kg^−1^. Phenolic compound accumulation was affected by the HM exposure time and corresponded to each HM and its concentration. Future research should analyze whole plants to determine the capabilities of *Buddleja cordata* to accumulate abnormally high amounts of HM and to evaluate the physiological impact of changes in the accumulation of phenolic compounds.

## 1. Introduction

Plants are evolutionarily adapted to environmental changes, allowing them to absorb heavy metals (HMs) present at high levels in soils via well-coordinated molecular mechanisms through morphological, physiological, and metabolic adaptations [1,2,3]. All plant species presumably have a basal heavy metal (HM) tolerance provided by a complex system of uptake/efflux, transport/sequestration, and chelation processes. However, the characteristics of these processes vary among species, and plant species can be categorized as hyperaccumulators or non-accumulators [4]. Most plants are non-accumulators and tend to avoid metal uptake, which is the simplest strategy for tolerating high HM exposure. However, HMs can induce phytotoxicity, which presents as altered metabolic activity, growth reduction, and low biomass production [3,4]. The capability of plants to accumulate abnormally high amounts of metals (at levels 100- to 1000-fold greater than those typically measured in non-accumulator plants) in their aboveground organs is represented by the term “hyperaccumulation” [5,6]. Hyperaccumulator species are characterized by their tolerance of HMs and detoxification mechanisms. For example, the overexpression of the transport protein systems required to enhance sequestration, the chelation of metals, and the fast and effective repair of damage caused by HMs may include changes in antioxidant defenses, the expression of heat shock proteins, and the induction of secondary metabolites (e.g., phenolic compounds). Chelation with ligands may also occur, enabling HM ions to be transported and sequestered in different plant tissues [4,5,7]. Phenolic compounds can act as ligands that form complexes with metals. This complexation may have indirect effects such as facilitating cell penetration; changing the redox state of the metal, consequently altering its bioavailability; and producing reduction activity in redox-active metals. Phenolic compounds can also act as antioxidants during the oxidation process due to their radical-scavenging activities based on hydrogen or electron donation, which results in stable phenoxyl radicals [8].

Hyperaccumulator species have been found in at least 52 families, including Brassicaceae, Fabaceae, Euphorbiaceae, Asteraceae, Lamiaceae, and Scrophulariaceae [2,5,6]. In addition, several hyperaccumulator species grow in soils rich with metals, such as ultramafic soils. These soils are generally infertile and edaphically stressful for the growth and survival of most plant species. However, ultramafic species have evolved adaptations to tolerate these stressful conditions [9], such as some species belonging to the *Buddlejaceae* genus (Scrophulariaceae). *Buddleja asiatica* was reported to be a lead hyperaccumulator [10,11], while *B. paniculata* and *B. scordioides* were reported to be lead accumulators [11,12]. Although *Buddleja cordata* Humb. Bonpl. and Kunth is distributed in a wide range of habitats in Mexico, including habitats with infertile soils [13,14], the most recent study to determine its hyperaccumulation potential was published by Navarrete et al. [14]. In that work, the ultramafic vegetation of central and southern Mexico was assessed to identify Ni hyperaccumulator species. Although *B. cordata* presented low Ni accumulation (10 mg kg^−1^), Ca, Co, Fe, Mg, Mn, and Zn were accumulated at concentrations of 6889, 1.2, 1788, 4481, 22, and 13.6 mg kg^−1^, respectively, in the plant’s leaves when grown in an ultramafic soil characterized by high Mg, Fe, Cr, Co, and Ni concentrations and Ca macronutrient deficiencies [14]. Leaf litter samples of *B. cordata* grown near Mexico City, which is highly polluted, indicated an increased mobilization of some HMs in the soil (essential metal nutrients Cu, Fe, Mn, and Zn and nonessential metal nutrients Al, Cd, and Pb) based on hydrosoluble phenolic substances [15]; the authors argued that these compounds might act as chelators.

Research on hyperaccumulators has increased worldwide because such species represent an environmentally friendly and inexpensive way to restore HM-polluted soils through phytoremediation [2,16,17]. Moreover, plant cell cultures are a useful tool used to investigate plant responses to metal pollution. These cultures allow one to control environmental and nutritional conditions, enabling researchers to estimate the intrinsic phytoremediation potential of plants. In addition, the use of plant cultures reduces the time required for experiments because they rely on continuously propagated vegetal material rather than whole plants [18].

In previous work with *B. cordata*, we established a cell culture producer of phenolic compounds such as phenolic acids (hydroxycinnamic acids), linarin (flavonoid), and verbascoside (phenylpropanoid glycoside) [19]. This culture was characterized by its growth and phenolic compound concentration since these phenolic compounds are related to the use of this species in Mexican traditional medicine; its medicinal uses are related to wounds or skin ailments, rheumatism, liver disorders, headaches, gastrointestinal infections, muscle cramps, nasal hemorrhages, and diuretic and kidney treatments, of which their therapeutic effects have been mainly attributed to phenolic compounds [19]. Thus, this type of cell culture can be used to investigate the ability of *B. cordata* to tolerate HMs. The present study aimed to determine the effects of different concentrations of Cu, Fe, Mn, and Zn on growth, HM accumulation, and phenolic compound accumulation to study the HM tolerance of the *B. cordata* cell suspension culture.

## 2. Results

### 2.1. Tolerance and Accumulation of HMs in a B. cordata Cell Suspension Culture Grown in a Culture Medium Enriched with Cu, Fe, Mn, and Zn

After HM exposure, the *B. cordata* cell suspension culture showed significant changes in its growth parameters (cell viability and growth index), and its environment (pH, and total sugar content of the culture medium) caused by the type of HM and its concentration. The highest concentration tested for each HM (0.25 mM Cu, 1.5 mM Fe, 3.0 mM Mn, and 2.0 mM Zn) had the strongest effect on the growth parameters and yielded the most significant growth inhibition (with growth indexes of 0.21, 0.44, 0.34, and 0.24, respectively); the lowest cell viability percentages (20.5, 12.86, 10.34, and 12.2, respectively); the lowest pH values (4.22, 4.43, 4.11, and 4.34, respectively); and the highest total sugar contents (28.13, 27.56, 27.88, and 28.31 g L^−1^, respectively) in the culture medium (Table 1). In the remaining HM treatments, the cells were able to grow, presenting growth index values > 3.29. Notably, the growth index values in several HM treatments (0.03–0.10 mM Cu, 0.25–0.5 mM Fe, 0.5–2.0 mM Mn, and 0.5–1.25 mM Zn) were not significantly different from those in the control (growth index of 6.46). In addition, the cell viability, total sugar content, and pH values in these treatments were slightly or not different from those in the control (81.33%, 5.57 g L^−1^, and 6.38, respectively; Table 1).

**Figure 1:**
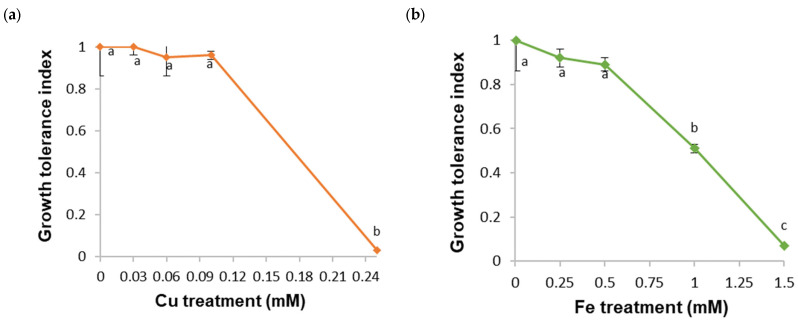

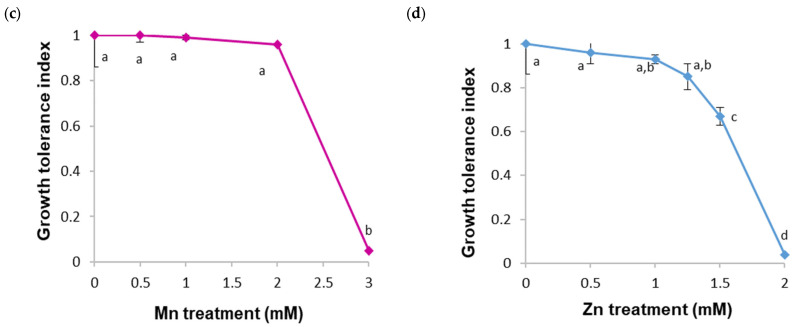
Effects of (**a**) Cu, (**b**) Fe, (**c**) Mn, and (**d**) Zn at different concentrations on the growth tolerance index of the *B. cordata* cell suspension culture grown for 18 days in the culture medium enriched with heavy metals. All results are shown as the mean ± SD. The same lowercase letter indicates no significant difference at a 5% significance level.

All these results show that the *B. cordata* cell suspension culture was unable to tolerate the highest HM concentrations tested, inhibiting growth and leading to death, acidification, and the nonconsumption of sucrose in the culture medium. Indeed, growth tolerance index values lower than 0.1 were observed (0.03, 0.07, 0.05, and 0.04 for 0.25 mM Cu, 1.5 mM Fe, 3.0 mM Mn, and 2.0 mM Zn, respectively; Figure 1a–d). Therefore, HM accumulation in the *B. cordata* cell suspension culture was not determined at the highest HM concentrations tested. However, the *B. cordata* cell suspension culture tolerated the remaining HM concentrations tested and grew despite the presence of HMs at considerably higher concentrations (600- to 2000-fold greater for Cu, 5- to 20-fold greater for Fe, 10- to 40-fold greater for Mn, and 33.3- to 100-fold greater for Zn) than those in the control treatment (0.00005 mM Cu, 0.05 mM Fe, 0.05 mM Mn, and 0.015 mM Zn). For all other tested HM concentrations, the growth tolerance index values were higher than 0.51 (Figure 1a–d).

**Figure 2:**
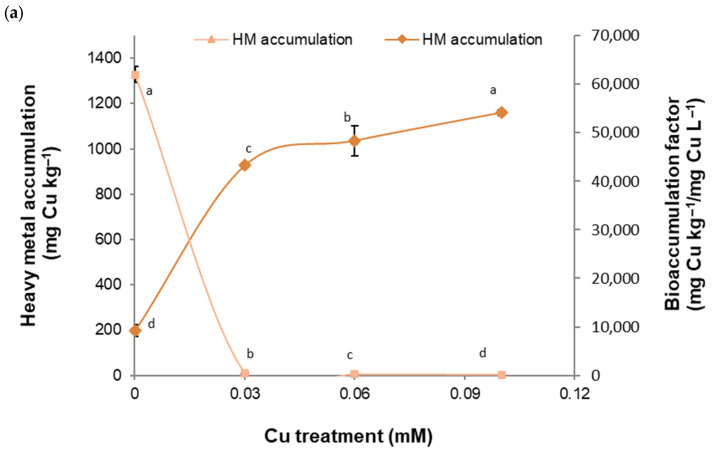

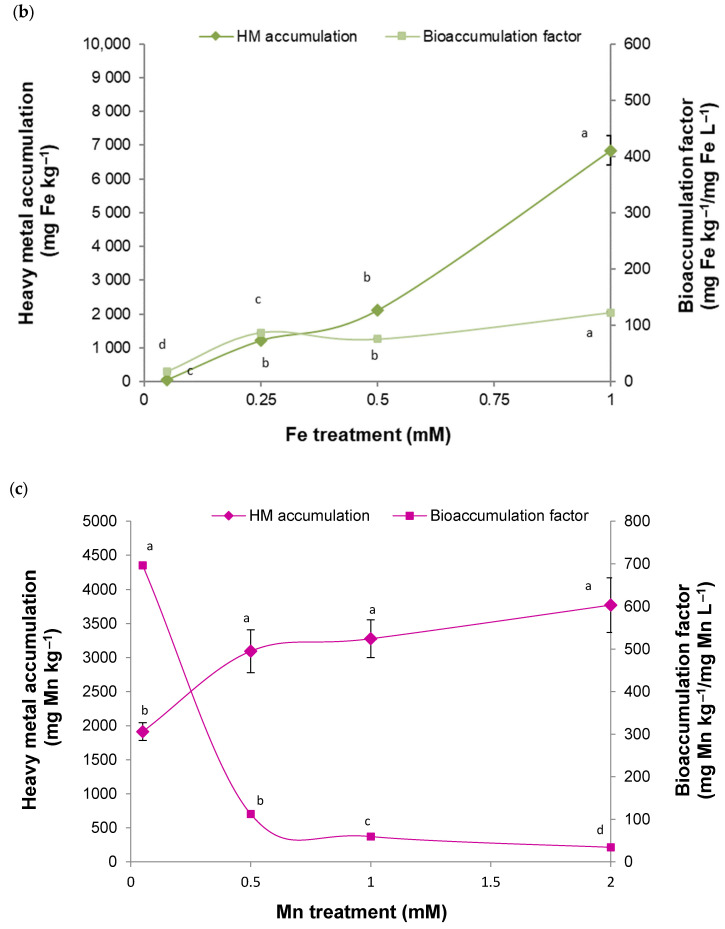

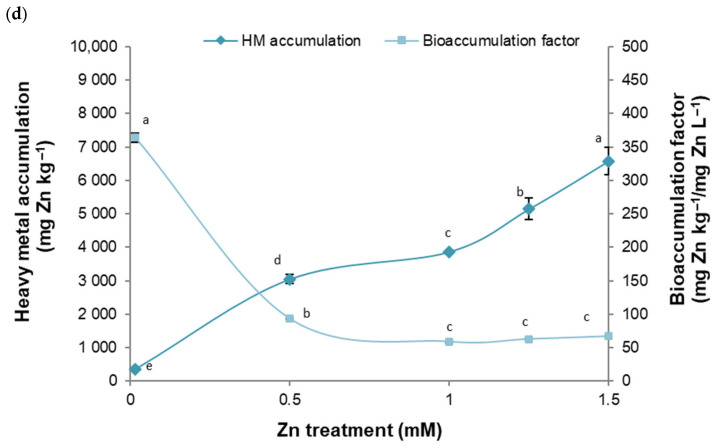
Heavy metal accumulation and bioaccumulation factors of Cu, Fe, Mn, and Zn in the *B. cordata* cell suspension grown for 18 days in a culture medium enriched with the heavy metals at different concentrations: (**a**) 0.03 to 0.25 mM Cu; (**b**) 0.25 to 1.5 mM Fe; (**c**) 0.5 to 3.0 mM Mn; and (**d**) 0.5 to 2.0 mM Zn. The concentrations of the heavy metals in the control treatment were 0.00005 mM Cu, 0.05 mM Fe, 0.05 mM Mn, and 0.015 mM Zn. All the results are shown as the mean ± SD. The same lowercase letter indicates no significant difference at a 5% significance level for a particular response variable.

Exposing the *B. cordata* cell suspension culture to the four HMs provoked a significant increase in HM accumulation (Figure 2a–d). For Cu, Fe, and Zn, HM accumulation was directly influenced by the HM concentration (Figure 2a,b,d). The highest accumulation values for each HM were 1160 mg Cu kg^−1^, 6845 mg Fe kg^−1^, 3770 mg Mn kg^−1^, and 6581 mg Zn kg^−1^ at 0.1 mM Cu, 1 mM Fe, 2 mM Mn, and 1.5 mM Zn, respectively (Figure 2a–d). In addition, the ability of the *B. cordata* cell suspension culture to take up HMs from the culture medium, expressed as the bioaccumulation factor, significantly decreased under Cu, Mn, and Zn exposure and was inversely influenced by the HM concentration. However, we observed opposite effects under Fe exposure (Figure 2a–d).

### 2.2. Total Phenolic and Verbascoside Content Changes in the B. cordata Cell Suspension Culture Grown in a Culture Medium Enriched with Cu, Fe, Mn, and Zn

**Figure 3:**
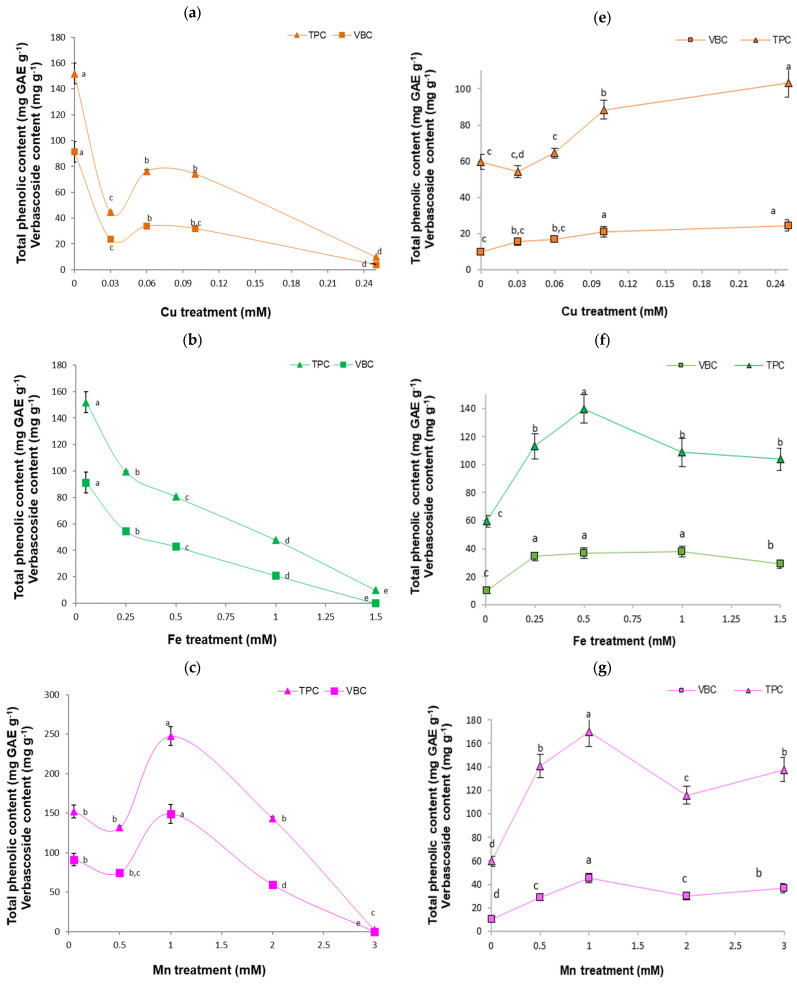

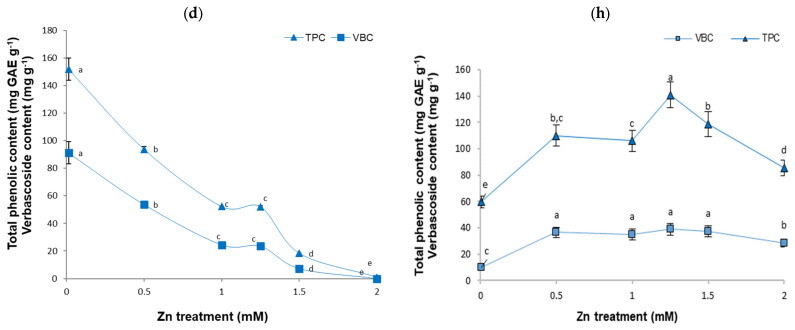
Phenolic compound accumulation (total phenolic and verbascoside contents) in the *B. cordata* cell suspension culture grown for 18 and 1 day(s) in a culture medium enriched with heavy metals at different concentrations. At 18 and 1 day(s): (**a**,**e**) 0.03 to 0.25 mM Cu; (**b**,**f**) 0.25 to 1.5 mM Fe; (**c**,**g**) 0.5 to 3.0 mM Mn; and (**d**,**h**) 0.5 to 2.0 mM Zn. The concentrations of heavy metals in the control treatment were 0.00005 mM Cu, 0.05 mM Fe, 0.05 mM Mn, and 0.015 mM Zn. All results are shown as the mean ± SD. The same lowercase letter indicates no significant difference at a 5% significance level for a particular response variable.

The exposure time of the *B. cordata* cell suspension culture to the four HMs yielded significant changes in phenolic compound accumulation. In general terms, HM exposure over 18 days of culture decreased the total phenolic and verbascoside contents, which were inversely affected by the HM level, compared with the control; however, the opposite occurred at 1.0 mM Mn (Figure 3a–d). Total phenolic and verbascoside content values were found to be the lowest under the highest tested metal concentrations, with values as low as zero (Figure 3a,b). In contrast, treating the *B. cordata* cell suspension culture with 1.0 mM Mn yielded the highest accumulation of phenolic compounds, with higher values observed for the total phenolic (248 mg gallic acid equivalent g^−1^ [mg GAE g^−1^]) and verbascoside (224 mg g^−1^) contents compared to the control treatment (152 mg GAE g^−1^ and 137 mg g^−1^, respectively) (Figure 3a,b). After 1 day of HM exposure, the values of the total phenolic and verbascoside were higher than those of the control, which was an effect that depended on each HM and its concentration (Figure 3e–h).

## 3. Discussion

Some HMs such as Cu, Fe, Mn, and Zn are essential nutrients for plants. These metals play vital functions in plant metabolism, e.g., serving as integral components in several enzymes that participate in redox reactions and nucleic acid metabolism [3]. These metals are normally found in plants at low concentrations (6, 100, 50, and 20 mg kg^−1^ for Cu, Fe, Mn, and Zn, respectively) [20]. Consequently, HMs are required in low amounts, and exposure to high concentrations can result in toxicity [3]. However, Cu and Fe hyperaccumulator species can harbor at least 1000 mg kg^−1^ of these metals, while Mn and Zn hyperaccumulators can contain 10,000 mg kg^−1^ [6], without showing toxicity.

The results of this work suggest that *B. cordata* cells have tolerance traits that allow them to accumulate Cu, Fe, Mn, and Zn and thus resist toxicity and grow despite the HM concentrations being higher than those in the control. This tolerance was indicated by the high growth index, growth tolerance index, and cell viability values, as well as the low total sugar content values in the residual medium. Together, these results indicate a high consumption of the sugar substrate required for growth. At the cellular level, plants can feature a range of detoxification mechanisms that activate to prevent the accumulation of toxic HM concentrations within the cell, such as the involvement of the plasma membrane in reducing the uptake of HM; chelation in the cytosol by ligands, resulting in complex formation; or the transport of HM/HM complexes to vacuoles [21]. In this study, the plasma membranes of the *B. cordata* cells, as a detoxification mechanism, may have reduced the uptake of Cu and Mn since HM accumulation greatly increased under the first concentration tested, followed by a slight increase that plateaued as the concentration increased. However, for Fe and Zn, the HM accumulation increased as the HM concentration increased. In this case, the plasma membranes of the *B. cordata* cells likely did not reduce uptake, and HM chelation may have occurred. HM conjugates can be formed with amino acids, vitamins, organic acids, glutathione, phytochelatins (PCs), and metallothioneins [4,5,7,22,23]. Indeed, Cu and Zn have shown to induce the formation of PC complexes [24,25]. The enzyme responsible for PC synthesis is a constitutive cytoplasmic enzyme activated through exposure to several HMs, such as Zn. This enzyme has been reported to be essential in detoxifying large amounts of Zn by promoting its accumulation [26]. *B. cordata* cells may also form HM complexes that allow the cells to accumulate HMs.

Furthermore, the antioxidant mechanisms of *B. cordata* cells may allow the cells to accumulate HMs and grow. A common consequence of HM poisoning is the enhanced production of reactive oxygen species (ROS) that expose cells to oxidative stress, leading to RNA and DNA synthesis errors, enzyme activity inhibition, lipid peroxidation, and the blocking and displacement of essential functional groups in biomolecules [5,27]. The tolerance of hyperaccumulator species involves enhanced antioxidant (enzymatic and nonenzymatic) defense mechanisms to counteract this oxidative stress [5,28]. Notably, the antioxidant enzymatic mechanisms of plants are considered an important defense against the oxidative stress induced by HMs [3]. The primary enzymes implicated in ROS scavenging are superoxide dismutase, catalase, and peroxidase. Superoxide dismutase is the first line of defense against reactive oxygen species, followed by catalase and peroxidase [29]. However, after a prolonged period of HM exposure, under the highest tested concentrations of each HM, the antioxidant mechanism of the *B. cordata* cells was likely unable to counteract the oxidative stress provoked by each HM, thereby damaging the integrity of the cells and leading to a low growth index, growth tolerance index, and cell viability values, as well as high total sugar contents. The integrity of the cells may have been affected by the peroxidation of lipids from the plasma membrane [27], which provoked the release of organic acids including phenolic acids. These conditions resulted in the lowest pH values in the culture medium. Moreover, phenolic compounds, including verbascoside, likely contributed to the nonenzymatic antioxidant mechanisms in *B. cordata* cells, which counteracted the adverse effects of high HM concentrations, as phenolic compound accumulation significantly increased after 1 day of HM exposure. An antioxidant (nonenzymatic) system consisting of metabolites with low molecular weights, such as flavonoids, phenolic acids, and phenylpropanoids, has been recognized to be effective in directly scavenging the reactive oxygen species produced by the presence of HMs [30]. Likewise, the phenolic compounds produced by *B. cordata* cells likely contributed to the transport of Fe since the HM accumulation and bioaccumulation factors both increased as the Fe concentration increased. Some plants have been reported to release organic anions, such as phenolic compounds, to increase HM solubility by forming chelates [31,32]. This phenomenon has been shown to improve Fe transport [31,32] and confer an additional ability to tolerate and grow under highly toxic metal concentrations in hyperaccumulator species [4,33]. Different experimental models have demonstrated that verbascoside exerts antioxidant activity by scavenging free radicals or chelating the HMs (Cu^2+^, Fe^2+^, and Fe^3+^) that mediate oxidation [34,35]. This compound contributes to protecting plants from the DNA damage from hydroxyl radicals induced by Fe^2+^ and Fe^3+^ since it can form complexes with HMs (formation constants of 1021.03 and 1031.94 M^−2^ for Fe^2+^ and Fe^3+^, respectively) [34].

The results of this work could serve as a useful approach for continuing research on *B. cordata* as a species with tolerance traits that could be useful for phytoremediation purposes. Indeed, in vitro cultures of several plant species were demonstrated to be useful tools for researching topics in phytoremediation. A tolerant callus of *Brassica* spp., taxonomically similar to *Brassica juncea* and a hyperaccumulator crop used for phytoremediation in polluted soil, has shown a high HM content in cells, with 3.83 mg Zn kg^−1^ and 2.91 mg Mn kg^−1^ under 0.24 mM Zn and 0.8 mM Mn, respectively [36]. The callus presented reductions of 33 and 30%, respectively, in fresh weight compared with that in the control [36]. The growth of *Sesbania drummondii* cell cultures (a species used in phytoremediation) was negligible at 500 mg Cu L^−1^ (~7.87 mM). However, under 100 mg Cu L^−1^ (~1.58 mM), the cells were able to grow and accumulate 3000 mg Cu kg^−1^ [31]. In both studies, the activity of antioxidant enzymes was a feature associated with metal tolerance [36,37]. Treating callus cultures of *Acer pseudoplatanus* (a tree that grows in soils polluted with metals) obtained from several populations with 0.08 mM Cu or 1.91 mM Zn yielded different levels of HM accumulation (100–400 mg Cu kg^−1^ and 6000–10,000 mg Zn kg^−1^) [38]. A cell suspension culture of *Thlaspi caerulescens* (a Zn/Cd hyperaccumulator species) exhibited no changes in growth after 10 days of treatment with Zn concentrations from 30 to 1500 µM, while the HM accumulation increased from 30 to 200 µmol g^−1^ (equivalent to 1962 to 13,080 mg Zn kg^−1^) [39]. Treating in vitro root cultures of *Scirpus americanus* with 1.8 mg Mn L^−1^ (~0.033 mM) did not affect growth and yielded 5000 mg Mn kg^−1^. This organism was found to be a Mn accumulator [40]. Tolerant somaclones developed from the calli of some indica rice varieties grew at 50–400 ppm Fe (~0.89–7.15 mM). Callus growth decreased under higher Fe concentrations until complete necrosis occurred at 400 ppm [41]. Studies have also been performed on HMs that are not essential nutrients for plants. For example, Bernabé-Antonio et al. [42] demonstrated high accumulations of Cr and Pb contents in a cell culture of *Jatropha curcas*. This species was shown to have significant potential for HM phytoremediation [42]. Thus, according to the results for the *B. cordata* cells in the present study and in the context of previous publications, *B. cordata* may possess outstanding tolerance mechanisms that could be useful for phytoremediation. This ability might be related to the membership of *B. cordata* in the *Buddlejaceae* genus [10,11,12]. In addition, several hyperaccumulator species grow in soils rich with metals, such as ultramafic soils [9], in which *B. cordata* also grows [14].

Plant metal tolerance is typically specific to particular metals [38], and the results found in this work show that *B. cordata* may be tolerant to several metals. *Bacopa monnieri* showed high tolerance to Mn > Cr > Cu > Cd > Pb, as no visible phytotoxic symptoms were observed after two months of HM exposure (0.003–0.160 mM for each HM) [43]. In vitro seedlings of *Prosopis laevigata* showed a strong ability to tolerate and accumulate Cr, Cd, Pb, and Ni [44,45]. The tolerance capabilities of the *B. cordata* cell culture against must be tested different HM ions from those tested in this work, including those of nonessential HMs, since the transport of metal ions occurs regardless of whether or not they are essential [23]. Several plant species can grow in metalliferous soils, and these rare species have valuable potential for phytoextraction [1]. Thus, this work contributes to exploring the potential of *B. cordata* for further research on phytoremediation. Moreover, the results of this work highlight the care that must be taken when *B. cordata* is used for medicinal purposes because the plant may have accumulated high amounts of HMs (essential or nonessential metal nutrients) and may thus represent a serious risk to human health. Higher amounts of some HMs than those required to maintain good health can be toxic or dangerous [46]. Therefore, avoiding exposure to high HM concentrations is a protective measure necessary to support human wellbeing [46]. In addition, the high amounts of the four HMs accumulated by the *B. cordata* cells and their impacts on phenolic compound accumulation after 1 day or 18 days of HM exposure could have both ecological and biotechnological implications. Ecological studies should be undertaken to determine the evolutionary value of high HM accumulation as an advantage over pathogens or herbivores. It was previously hypothesized that hyperaccumulation provides a defense against pathogens or herbivores [47]. Cell suspension cultures of plants represent a biotechnological tool for producing bioactive secondary metabolites, and elicitation can be used to increase the production of secondary metabolites [19]. Elicitation is a strategy used to promote the increased biosynthesis and accumulation of secondary metabolites, through activating the defense system against biotic or abiotic stress [24]. In this work, we demonstrated that phenolic compound accumulation was significantly increased after 1 day of HM exposure compared with that in the control, so future research should be carried out to identify the specific secondary metabolites, in addition to verbascoside, with increased accumulation. Metals act as signal molecules or abiotic elicitors that stimulate the production of secondary metabolites such as alkaloids, terpenoids, and phenolic compounds as a defense response to stressful stimuli [20,24,48,49]. Treating *Panax ginseng* root cultures with 5–50 µM Cu affected the ginsenoside metabolite content; ginsenoside content was stimulated from 5–25 µM Cu, but under 50 µM Cu, it was decreased [50]. The maximum increase in flavonoid production in *Trifolium pratense* suspension cultures occurred after 7 days of copper application (0.1 mM) [51]. Producing high yields of bioactive secondary metabolites through in vitro plant cultures is highly desirable, with several potential strategies including elicitation and optimizing the culture medium [52].

## 4. Materials and Methods

### 4.1. Cell Suspension Culture

In a previous study using a *B. cordata* cell suspension culture, the outstanding growth parameters were a maximum biomass of 12.9 g L^−1^ and growth index of 8.14 after 14 days of culturing, cell viability > 80% during the exponential phase of growth, and total substrate consumption at 30 days of culturing (measured through total sugar content). The verbascoside content was associated with growth and reached its maximum level (116.36 mg g^−1^ dry weight (DW), equivalent to 1.44 g L^−1^ for verbascoside) during the stationary phase at 16–22 days of culturing [19]. Based on the growth profile of the culture, this work was carried out over 18 days of HM exposure to estimate the effects on growth and phenolic accumulation.

An in vitro cell culture of *B. cordata* was donated to the Universidad Autonoma Metropolitana-Iztapalapa Campus (UAM-I), México D.F., México, as a suspension culture and proliferated according to Estrada-Zúñiga et al. [19]. Briefly, the basal culture medium (BCM) consisted of half-strength Murashige and Skoog medium [53] with 3% weight/volume (*w*/*v*) sucrose, 0.45 µM 2,4-dichlorophenoxyacetic acid, 2.32 µM kinetin, 100 mg L^−1^ citric acid, and 150 mg L^−1^ ascorbic acid. Cell suspension cultures were subcultured ten times to proliferate their biomass [19], which was required for the HM bioassays. The cell suspension cultures under proliferation were incubated in a gyratory shaker at 110 rpm with a photoperiod of 16 h light (30 µmol m^−2^ s^−1^) at 26 ± 2 °C.

### 4.2. HM Bioassays

The HM bioassays involved growing *B. cordata* cells in BCM enriched with Cu, Fe, Mn, and Zn. The biomass from the last subculture was used to inoculate 125 mL Erlenmeyer flasks (1.5 g of fresh weight per flask) containing 25 mL of the culture medium. This process involved the addition of the HMs for the enrichment of culture medium until achieving the following concentrations: 0.03, 0.06, 0.1, and 0.25 mM Cu; 0.25, 0.50, 1.0, and 1.5 mM Fe; 0.5, 1.0, 2.0, and 3.0 mM Mn; and 0.5, 1.0, 1.25, 1.5, and 2.0 mM Zn. The BCM contained these HMs at micromolar concentrations (0.05 µM Cu, 50 µM Fe, 50 µM Mn, and 15 µM Zn). Additionally, we carried out a preliminary experiment to establish the maximum concentration tested for each HM based on the concentration that caused growth inhibition. Stock solutions (20 mg mL^−1^) of CuSO_4_·5H_2_O, FeSO_4_·7H_2_O, ZnSO_4_·7H_2_O, and MnSO_4_·H_2_O (Baker Analyzed, Phillipsburg, NJ, USA) were used as sources of the HMs. Corresponding aliquots of each stock solution were added to the BCM. The control treatments were as follows: (i) BCM and (ii) BCM with SO_4_^2−^ at the same concentrations used in the HM treatments (BCM-SO_4_), with a (NH_4_)_2_SO_4_ stock solution (20 mg mL^−1^) used as the source of SO_4_^2−^. This control was used because sulfur is an essential nutrient for plants, and its enrichment in the culture medium was hypothesized to have effects on *B. cordata* cells. However, when the response variables were determined for the *B. cordata* cells in both control treatments (BCM and BCM with SO_4_^2−^), the results were not significantly different. Thus, these treatments are both referred to as the control treatment in the present study, and data from these treatments were pooled. Deionized water was used to prepare stock solutions of HM salts and the medium. When all components of the medium were added, the pH was adjusted to 5.8 with NaOH 0.1 M, and the samples were autoclaved at 121 °C for 18 min. Each treatment consisted of two Erlenmeyer flasks with three replicates (n = 6).

Once biomass was inoculated in the 125 mL Erlenmeyer flasks containing 25 mL of sterile medium enriched with the HM, the resulting cultures were incubated in the same conditions described above for proliferation purposes. After 18 days of culturing, several response variables were measured to estimate the tolerance of cells exposed to HMs. For this purpose, biomass from the HM bioassays was harvested via vacuum filtration while washing with 100 mM EDTA, dried in an oven at 60 °C, and weighed. The residual medium was used to determine the pH value and total sugar content of the biomass. Cell viability, total sugar content, and growth index measurements were carried out as described by Estrada-Zúñiga et al. [19]. Different system responses in plant cell suspension cultures (e.g., growth) can be used to study whether the plant cells are subjected to factors that cause stress [54]. Additionally, the tolerance of the cells was calculated based on the growth tolerance index using Equation (1) according to Rout et al. [36], with some modifications:

(1) $$Growth\;tolerance\;index=\frac{Growth\;index\;of\;cells\;grown\;in\;culture\;medium\;with\;HM}{Growth\;index\;of\;cells\;grown\;in\;culture\;medium\;without\;HM}$$

The growth index was estimated based on the DW as follows:

(2) $$Growth\;index=\frac{Final\;biomass\;(mg\;DW)-initial\;biomass\;(mg\;DW)}{initial\;biomass\;(mg\;DW)}.$$

### 4.3. Determination of the HM Content in Cells

After 18 days of culturing, cells (100 mg DW) harvested from the HM-exposure treatments were powdered and digested with 5 mL of concentrated HNO_3_ in a microwave oven (CEM Mars5, CEM Corporation, Charlotte, NC, USA). Then, the volume of each digested sample was adjusted to 10 mL with deionized water, and the samples were retained in high-density polyethylene flasks. An atomic absorption spectrometer (Varian Spectra AA-220 FS, Varian, Belrose, Australia) was used to analyze the HM content in the cells from the digested samples. Calibration curves (0.5 to 1.5 mg mL^−1^ for Cu; 0.5 to 2.5 mg mL^−1^ for Fe; 0.5 to 3.5 mg mL^−1^ for Mn or Zn) were generated using HM standards corresponding to the analyzed HM (Baker Analyzed, Phillipsburg, NJ, USA). The results were expressed as mg of HM per kg of DW biomass (mg HM kg^−1^). A solution of 0.1 N HNO_3_ was used to wash the glassware and equipment before use. Additionally, the bioaccumulation factor (determined only for treatments showing a growth index > 1) was estimated according to Bernabé-Antonio et al. [55], as follows:

(3) $$Bioaccumulation\;factor=\frac{HM\;content\;in\;cells\;grown\;in\;culture\;medium\;enriched\;with\;HM\;(mg\;HM/kg)}{HM\;content\;in\;culture\;medium\;(mg/L)}$$

### 4.4. Effect of HM on the Accumulation of Phenolic Compounds

The dry biomass from the HM exposure treatments of 18 days (60 mg DW) was extracted for 60 min with boiling MeOH (30 mL). All results were expressed on a per g basis of DW biomass. The total phenolic content was determined according to Vazquez-Marquez et al. [56]. The corresponding results were expressed as mg GAE g^−1^. All reagents were acquired from Sigma–Aldrich Co., Ltd., St. Louis and Burlington, MA, USA. The verbascoside content was determined with an Agilent Technologies 1100 series high-performance liquid chromatography system using a G1311A Quatpump (Alltech Co., Ltd., Nicholasville, KY, USA) equipped with an Econosil C18 column (4.6 mm × 250 mm, 5μ; Alltech Co., Ltd., Nicholasville, KY, USA) and a G1315B diode array detector (Alltech Co., Ltd., Nicholasville, KY, USA). The operating conditions of this system included a mobile phase of 2.0% (*v*/*v*) acetic acid solution (solvent A) and acetonitrile (solvent B), an injection volume of 20 µL, a flow rate of 1.0 mL min^−1^, and a detection wavelength of 330 nm. The system was run with a gradient program as follows: 10 min, 90% to 75% A, and 15 min, 75% to 60% A. Agilent 1100 Chemstation chromatography software (Rev. A.08.03; Agilent Technologies, Santa Clara, CA, USA) was used to acquire data from the detector. A calibration curve (80–240 µg mL^−1^) was created using the verbascoside standard (Extrasynthese, Genay, L, France). The results were expressed as mg of verbascoside per g of DW biomass (mg g^−1^). Peak areas at the corresponding standard retention times were used for detecting, identifying, and quantifying verbascoside in the samples. Each sample was injected twice (n = 2). In addition, cells were also grown for 1 day in a medium enriched with Cu, Fe, Mn, and Zn treatments to determine the effect of the HMs on phenolic compound accumulation. Thus, the same procedure described in this section was carried out.

### 4.5. Statistical Analysis

The results for the response variables (growth index, growth tolerance index, cell viability, total sugar content, pH, HM accumulation, bioaccumulation factor, total phenolic content, and verbascoside content) were analyzed using one-way ANOVA, followed by the Tukey–Kramer post hoc test for multiple comparisons. NCSS version 12 software (East Kaysville, UT, USA) was used for all statistical analyses. A *p* value of less than 0.05 was uniformly considered to indicate significant differences.

## 5. Conclusions

The *B. cordata* cell suspension culture presented strong HM tolerance traits that allowed the cells to grow and accumulate high amounts of Cu, Fe, Mn, and Zn (1160 mg Cu kg^−1^, 6845 mg Fe kg^−1^, 3770 mg Mn kg^−1^, and 6581 mg Zn kg^−1^). This species may have hyperaccumulator potential for HMs as follows, from least to most significant: Mn < Zn < Cu < Fe. This potential should be evaluated in future research with whole *B. cordata* plants. In addition, future research should determine the ecological impact of a possible decrease in the accumulation of phenolic compounds in whole plants growing in heavy-metal-polluted soils. *B. cordata* cell suspension cultures exposed to HMs for 1 day may represent a useful biotechnological tool for enhancing the accumulation of bioactive phenolic compounds in this species.

## Figures and Tables

**Table 1:** Effects of Cu, Fe, Mn, and Zn enrichment on the growth and culture medium of the *B. cordata* cell suspension grown for 18 days.

Heavy Metal Treatment (mM)	Growth		Culture Medium	
	Cell Viability (%)	Growth Index	pH	Total Sugar Content (g L^−1^)
Copper				
Control (0.00005)	81.33 ± 1.58 ^a^	6.46 ± 0.88 ^a^	6.38 ± 0.05 ^b^	5.57 ± 0.40 ^b^
0.03	77.00 ± 3.54 ^a^	6.43 ± 0.27 ^a^	6.44 ± 0.05 ^b^	5.21 ± 0.72 ^b^
0.06	73.25 ± 0.65 ^b^	6.11 ± 0.58 ^a^	6.66 ± 0.02 ^a^	4.34 ± 0.61 ^b^
0.10	72.40 ± 1.15 ^b^	6.23 ± 0.15 ^a^	6.63 ± 0.10 ^a^	4.44 ± 0.54 ^b^
0.25	20.50 ± 0.78 ^c^	0.21 ± 0.01 ^b^	4.22 ± 0.03 ^c^	28.13 ± 1.11 ^a^
Iron				
Control (0.05)	81.33 ± 1.58 ^a^	6.46 ± 0.88 ^a^	6.38 ± 0.05 ^a,b^	5.57 ± 0.40 ^c^
0.25	75.97 ± 2.10 ^b^	5.97 ± 0.24 ^a^	6.64 ± 0.05 ^a^	3.91 ± 0.15 ^c^
0.50	71.94 ± 1.82 ^b^	5.77 ± 0.18 ^a^	6.55 ± 0.10 ^a^	5.94 ± 0.02 ^c^
1.00	66.99 ± 0.84 ^c^	3.29 ± 0.14 ^b^	5.69 ± 0.01 ^c^	16.22 ± 2.01 ^b^
1.50	12.86 ± 1.26 ^c^	0.44 ± 0.02 ^c^	4.43 ± 0.01 ^d^	27.56 ± 0.40 ^a^
Manganese				
Control (0.05)	81.33 ± 1.58 ^a^	6.46 ± 0.88 ^a^	6.38 ± 0.05 ^a^	5.57 ± 0.40 ^b^
0.50	78.41 ± 1.85 ^a^	6.48 ± 0.19 ^a^	6.49 ± 0.04 ^a^	1.71 ± 0.22 ^c^
1.00	67.71 ± 2.53 ^b^	6.38 ± 0.09 ^a^	6.42 ± 0.09 ^a^	1.58 ± 0.29 ^c^
2.00	66.75 ± 2.25 ^b^	6.17 ± 0.01 ^a^	6.26 ± 0.04 ^b^	1.38 ± 0.33 ^c^
3.00	10.34 ± 0.25 ^c^	0.34 ± 0.04 ^b^	4.11 ± 0.04 ^b^	27.88 ± 1.26 ^a^
Zinc				
Control (0.015)	81.33 ± 1.58 ^a^	6.46 ± 0.88 ^a^	6.38 ± 0.05 ^a^	5.57 ± 0.40 ^d^
0.50	72.32 ± 2.44 ^b^	6.22 ± 0.35 ^a^	6.37 ± 0.06 ^a^	4.34 ± 0.46 ^d^
1.00	70.77 ± 1.83 ^b^	5.99 ± 0.13 ^a,b^	5.84 ± 0.06 ^b^	13.04 ± 1.79 ^c^
1.25	54.56 ± 0.41 ^c^	5.46 ± 0.41 ^a,b^	5.22 ± 0.03 ^c^	17.70 ± 0.80 ^b^
1.50	26.06 ± 1.40 ^d^	4.36 ± 0.24 ^b^	4.98 ± 0.04 ^d^	24.35 ± 1.66 ^a^
2.00	12.20 ± 2.54 ^e^	0.24 ± 0.02 ^c^	4.34 ± 0.02 ^e^	28.31 ± 2.48 ^a^

Data show the mean ± standard deviation (SD). For each heavy metal, the same lowercase letter after the mean ± SD value within a column indicates no significant difference at the 5% significance level. For each heavy metal, all the results are highlighted with a specific color: orange color was used for copper, green color was used for iron, pink color was used for manganese, and blue color was used for zinc.

## Data Availability

Data are contained within the article.
